# Effect of body mass index on the association between alcohol consumption and the development of chronic kidney disease

**DOI:** 10.1038/s41598-021-99222-y

**Published:** 2021-10-14

**Authors:** Yusaku Hashimoto, Takahiro Imaizumi, Sawako Kato, Yoshinari Yasuda, Takuji Ishimoto, Hiroaki Kawashiri, Akihiro Hori, Shoichi Maruyama

**Affiliations:** 1 Department of Nephrology, Nagoya University Graduate School of Medicine, 65 Tsuruma-cho, Showa-ku, Nagoya, Aichi 464-8550 Japan; 2 Takayama City Hall Public Health Department, Takayama, Gifu Japan; 3 Kumiai Kosei Hospital, Takayama, Gifu Japan

**Keywords:** Chronic kidney disease, Lifestyle modification

## Abstract

The influence of body mass or metabolic capacity on the association between alcohol consumption and lower risks of developing chronic kidney disease (CKD) is not fully elucidated. We examined whether the body mass index (BMI) affects the association between drinking alcohol and CKD. We defined CKD as an estimated glomerular filtration rate decline < 60 mL/min/1.73 m^2^ and/or positive proteinuria (≥ 1+). Participants were 11,175 Japanese individuals aged 40–74 years without baseline CKD who underwent annual health checkups. Daily alcohol consumption at baseline was estimated using a questionnaire, and the participants were categorized as “infrequent (occasionally, rarely or never),” “light (< 20 g/day),” “moderate (20–39 g/day),” and “heavy (≥ 40 g/day).” Over a median 5-year observation period, 936 participants developed CKD. Compared with infrequent drinkers, light drinkers were associated with low CKD risks; adjusted hazard ratios (95% confidence intervals) were 0.81 (0.69–0.95). Stratified by BMI (kg/m^2^), moderate drinkers in the low (< 18.5), normal (18.5–24.9), and high (≥ 25.0) BMI groups had adjusted hazard ratios (95% confidence intervals) of 3.44 (1.60–7.42), 0.75 (0.58–0.98), and 0.63 (0.39–1.04), respectively. Taken together, the association between alcohol consumption and CKD incidence was not the same in all the individuals, and individual tolerance must be considered.

## Introduction

Chronic kidney disease (CKD) with proteinuria and/or a lowered glomerular filtration rate (GFR) is a growing public health issue worldwide^1,2^. The number of CKD patients in Japan is 13.3 million, or 13% of the adult population^3^. CKD is an important condition as it is not only a risk factor for progression to end-stage kidney disease but also for the development of cardiovascular disease (CVD) and all-cause mortality^4–8^.

It is important to pay attention to modifiable risk factors such as lifestyle to prevent CKD development^9^. A systematic review identified modifiable lifestyle factors, including alcohol consumption, for the primary prevention of CKD^10^. This association was also observed across the entire range of alcohol consumption, with higher alcohol consumption associated with a lower risk of developing CKD. However, the systematic review demonstrated moderate heterogeneity, which suggested an inconsistent association between the amount of alcohol consumed and the risk of developing CKD. One of the studies in the systematic review showed that the risk of developing CKD decreased only in the group with an alcohol consumption < 20 g/day compared with that in the non-alcohol consuming group^11^. Moreover, recent cohort studies have shown that high alcohol consumption (≥ 60 g/day or ≥ 69.1 g/day) was associated with new-onset proteinuria^12,13^. However, most previous studies did not examine the association between alcohol consumption and CKD development relative to alcohol tolerance in each individual, which may have led to inconsistent results.

The incidence of hypertension (HT) and diabetes mellitus (DM), which are risk factors for CKD, has been reported to show a U-shaped or linear association with alcohol consumption^14–17^. Recent studies in Japan have shown that a higher body mass index (BMI) attenuates the risk of developing HT and DM associated with alcohol consumption^18–20^. However, only a few previous studies have examined this association with CKD development^21^, and no studies have evaluated it using stratification by BMI. Therefore, we hypothesized that a higher BMI has a more protective effect on the relationship between alcohol consumption and CKD development, whereas a lower BMI enhances the harmful effect on that relationship.

This study aimed to examine whether alcohol consumption is associated with CKD development in the general population and whether BMI modifies the association between alcohol consumption and the risk of CKD using data from health checkups.

## Results

### Subject population

**Figure 1:**
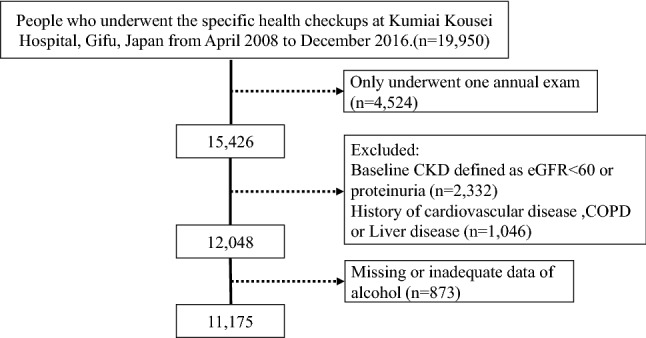
Flowchart of participant selection. Between April 2008 and December 2016, 19,950 participants underwent health checkups at Kumiai Kosei Hospital, of which 15,426 participants who underwent medical checkups at least twice were included in the study. Patients with chronic kidney disease at the time of the first visit (n = 2332) and those with a history of cardiovascular disease, chronic obstructive pulmonary disease, or liver disease (n = 1,046) were excluded, resulting in a total of 12,048 participants. Finally, 11,175 participants with sufficient information in the alcohol questionnaires were included in the analysis.

In this cohort, we enrolled 19,950 participants who underwent health checkups and excluded 4524 participants who underwent only one health examination: 2332 with prevalent CKD (estimated GFR [eGFR] < 60 mL/min/1.73 m^2^ or the presence of proteinuria) at baseline; 1046 who had CVD, chronic obstructive pulmonary disease (COPD), or liver disease; and 873 who had missing or inadequate data on alcohol drinking habits. CVD was defined as any of the following: myocardial infarction, coronary revascularization, heart failure, or stroke. Finally, the analytic cohort comprised 11,175 participants (Fig. 1).

### Baseline characteristics

Baseline characteristics according to alcohol consumption status are shown in Table 1. The median age was 62 years (interquartile range [IQR], 55–67 years). Compared to infrequent drinkers defined as occasionally, rarely, or never drinkers, the rest of the participants were more likely to be male, smokers, have higher blood pressure (BP), higher triglyceride (TG), and higher high-density lipoprotein cholesterol (HDL-C) levels, and lower low-density lipoprotein cholesterol (LDL-C) levels. During a median follow-up of 5 years (IQR, 2.9–7.6 years), there was a median of 5 visits (IQR, 3–8 visits), and a total of 936 patients developed CKD. Most participants underwent annual checkups, and the frequency of checkups was similar across groups.

**Table 1:** Baseline characteristics stratified by drinking status.

	Total (N = 11,175)	Infrequent drinkers (N = 6199)	Light drinkers < 20 g/day (N = 3157)	Moderate drinkers 20–39 g/day (N = 1162)	Heavy drinkers ≥ 40 g/day (N = 657)
Age	62 (55–67)	62 (56–67)	61 (53–66)	62 (53–67)	60 (50–65)
Sex male, n (%)	4494 (40%)	1368 (22%)	1544 (49%)	985 (85%)	597 (91%)
Current smoker, n (%)	2042 (18%)	787 (13%)	561 (18%)	393 (34%)	301 (46%)
BMI (kg/m^2^), mean [SD]	22.3 [3.1]	22.2 [3.2]	22.4 [2.9]	22.7 [2.9]	22.6 [2.9]
< 18.5, n (%)	994 (8.9)	674 (10.9)	222 (7.0)	56 (4.8)	42 (6.4)
18.5–24.9, n (%)	8196 (73.3)	4446 (71.7)	2363 (74.9)	894 (76.9)	493 (75.0)
≥ 25, n (%)	1985 (17.8)	1079 (17.4)	572 (18.1)	212 (18.2)	122 (18.6)
Antihypertensive drug, n (%)	1993 (18%)	982 (16%)	584 (18%)	254 (22%)	173 (26%)
Antihyperglycemic drug, n (%)	384 (3%)	228 (4%)	96 (3%)	39 (3%)	21 (3%)
Antihyperlipidemic drug, n (%)	1190 (11%)	824 (13%)	275 (9%)	58 (5%)	33 (5%)
eGFR (mL/min/1.73 m^2^), mean [SD]	78 [12]	77 [12]	78 [12]	79 [12]	81 [12]
Fasting blood glucose (mg/dL), mean [SD]	92 [18]	92 [17]	92 [17]	95 [17]	95 [19]
HbA1c (%), mean [SD]	5.7 [0.5]	5.8 [0.6]	5.7 [0.5]	5.7 [0.7]	5.6 [0.7]
LDL-C (mg/dL), mean [SD]	119 [30]	123 [29]	118 [29]	108 [29]	102 [31]
TG (mg/dL), median (IQR)	92(67–132)	92(68–128)	90(65–130)	96(68–144)	111(74–175)
HDL-C (mg/dL), mean [SD]	60 [14]	59 [14]	61 [14]	62 [15]	63 [16]
Systolic BP (mmHg), mean [SD]	125 [18]	123 [17]	125 [18]	130 [19]	132 [18]
Diastolic BP (mmHg), mean [SD]	75 [12]	74 [12]	75 [12]	79 [11]	80 [11]
Incidence of CKD, n (%)	936 (8.4)	484 (7.8)	249 (7.9)	119 (10.2)	84 (12.8)
Follow up period (year), median (IQR)	5.0 (2.9–7.6)	5.0 (3.0–7.7)	5.0 (2.7–7.6)	5.1 (3.0–7.3)	5.0 (3.0–7.1)
Number of checkups (visits), median (IQR)	5.0 (3.0–8.0)	5.0 (3.0–8.0)	5.0 (3.0–8.0)	5.0 (3.0–7.0)	5.0 (3.0–7.0)
Interval between visits (year / visit), median (IQR)	1.0 (1.0–1.1)	1.0 (1.0–1.1)	1.0 (1.0–1.0)	1.0 (1.0–1.1)	1.0 (1.0–1.1)

Data are presented as number (%) for categorical variables and mean [SD standard deviation] or median (IQR interquartile range) for continuous variables as appropriate.

*BMI* body mass index, *eGFR* estimated glomerular filtration rate, *LDL-C* low-density lipoprotein cholesterol, *TG* triglyceride, *HDL-C* high-density lipoprotein cholesterol, *BP* blood pressure, *CKD* chronic kidney disease.

### Alcohol consumption and CKD development

Table 2 shows the association between alcohol consumption and CKD development. The multivariable-adjusted hazard ratios (HRs) (95% confidence intervals; CIs) for CKD development were 0.81 (0.69–0.95) for light (< 20 g/day), 0.81 (0.65–1.00) for moderate (20–39 g/day), and 0.96 (0.74–1.23) for heavy (≥ 40 g/day) drinkers, relative to infrequent drinkers as a reference. No significant trend was observed across categories of alcohol consumption (*p* = 0.18 for trend). Regarding eGFR decline, alcohol consumption had no significant effect on the outcome. Regarding proteinuria, light and moderate drinkers had a lower risk with multivariable-adjusted HRs of 0.73 (95% CIs 0.60–0.90) and 0.73 (95% CI 0.56–0.94), respectively. The risk of developing proteinuria showed a significant inverse trend with an increase in alcohol consumption (*p* = 0.034 for trend).

**Table 2:** Result of the Cox proportional hazards model for the association between alcohol consumption and CKD development.

Amount of alcohol consumption	Incidence rate (per 1000 PY)	Age and sex adjusted model 1				Multivariate model 2^a^			
		HR	95% CI	*P* value	*P* trend^b^	HR	95% CI	*P* value	*P* trend^b^
**CKD (eGFR < 60 mL/min/1.73 m**^**2**^** and/or proteinuria)**									
Infrequent	16.2	Ref			0.32	Ref			0.18
< 20 g/day	16.6	0.81	0.69–0.95	0.01		0.81	0.69–0.95	0.01	
20–39 g/day	21.4	0.82	0.66–1.02	0.07		0.81	0.65–1.00	0.05	
≥ 40 g/day	27.2	1.01	0.79–1.30	0.91		0.96	0.74–1.23	0.74	
**eGFR decline to < 60 mL/min/1.73 m**^**2**^									
Infrequent	7.9	Ref			0.32	Ref			0.56
< 20 g/day	6.5	0.90	0.70–1.15	0.39		0.88	0.69–1.12	0.31	
20–39 g/day	6.4	0.96	0.65–1.41	0.84		0.92	0.62–1.34	0.65	
≥ 40 g/day	9.3	1.55	1.01–2.36	0.04		1.4	0.91–2.14	0.12	
**New-onset of proteinuria**									
Infrequent	9	Ref			0.036	Ref			0.034
< 20 g/day	10.2	0.72	0.58–0.88	0.001		0.73	0.60–0.90	0.003	
20–39 g/day	15	0.72	0.56–0.93	0.01		0.73	0.56–0.94	0.02	
≥ 40 g/day	19.2	0.84	0.63–1.13	0.25		0.82	0.61–1.11	0.21	

Outcomes were CKD (composite outcome of eGFR decline and/or new-onset of proteinuria) and eGFR decline and new-onset of proteinuria, respectively.

*CKD* chronic kidney disease, *eGFR* estimated glomerular filtration rate, *CI* confidence interval, *HR* hazard ratio, *PY* person-years.

^a^Multivariable adjustment included age, sex, eGFR, hypertension, diabetes mellitus, hyper lipidemia, body mass index, smoking status.

^b^*P* trend was derived from Cox proportional hazards regression models by treating alcohol consumption as a continuous linear term.

### Association between alcohol consumption and CKD stratified by BMI

Table 3 shows the association between alcohol consumption and CKD development stratified by BMI. In the high BMI group (BMI ≥ 25 kg/m^2^), the reduction in risk of CKD with alcohol consumption was more remarkable, while the multivariable-adjusted HRs (95% CI) for light drinkers were 0.80 (0.57–1.11), 0.63 (0.39–1.04) for moderate drinkers, and 0.61 (0.34–1.11) for heavy drinkers. There was a significant inverse trend of decreasing CKD risk with increasing alcohol consumption (*p* = 0.028 for trend). In contrast, higher HRs for risk of CKD with alcohol consumption were observed in the low BMI group (BMI < 18.5 kg/m^2^) group. The multivariable-adjusted HRs (95% CIs) were 1.26 (0.66–2.40) for light, 3.44 (1.60–7.42) for moderate, and 3.21 (1.23–8.37) for heavy drinkers. The risk of developing CKD showed a significant trend with an increase in alcohol consumption (*p* = 0.003 for trend). The association between alcohol consumption and the risk of CKD was modified by BMI (*p* = 0.005 for interaction). We also performed the following sensitivity analyses to examine the association between alcohol consumption and CKD incidence by using: (i) different definitions of drinking, (ii) the range of BMI categories, and (iii) the light drinkers as a reference. The drinking definition was based on the frequency of drinking or the amount of alcohol per consumption. The BMI categories were divided into three or four groups. (i) As in the main analysis, in the low BMI group, increased drinking frequency and alcohol amount per consumption were associated with CKD risk in the low BMI group, while the reverse was noted in the high BMI group (Supplementary Table S1). (ii) When BMI was divided into three or four categories, higher alcohol consumption was associated with a higher risk of CKD in the lower BMI categories compared with the higher BMI categories. (Supplementary Fig. S1). (iii) Using light drinkers as the reference category instead of infrequent drinkers attenuated the association between alcohol consumption amount and CKD incidence (Supplementary Table S2). In the low BMI group, the multivariable-adjusted HRs (95% CIs) were 0.80 (0.42–1.52) for infrequent, 2.74 (1.21–6.20) for moderate, and 2.56 (0.96–6.80) for heavy drinkers. In the high BMI group, the multivariable-adjusted HRs (95% CIs) were 1.25 (0.90–1.75) for infrequent, 0.63 (0.48–1.32) for moderate, and 0.77 (0.42–1.41) for heavy drinkers. However, it did not change the finding that the relationship between drinking and CKD risk varied according to BMI.

**Table 3:** Result of the Cox proportional hazards model for the association between alcohol consumption and CKD development stratified by BMI.

Amount of alcohol consumption	CKD				eGFR decline to < 60 mL/min/1.73 m^2^				New-onset of proteinuria			
	Multivariate model^a^				Multivariate model^a^				Multivariate model^a^			
	HR	95% CI	P trend^b^	P interaction^c^	HR	95% CI	P trend^b^	P interaction^c^	HR	95% CI	P trend^b^	P interaction^c^
**BMI < 18.5 kg/m**^**2**^				0.005				0.03				0.03
Infrequent	Ref		0.003		Ref		0.054		Ref		0.019	
< 20 g/day	1.26	0.66–2.40			0.79	0.28–2.23			1.77	0.76–4.14		
20–39 g/day	3.44	1.60–7.42			1.94	0.48–7.81			4.58	1.75–12.0		
≥ 40 g/day	3.21	1.23–8.37			5.86	1.42–24.2			2.66	0.75–9.40		
**BMI 18.5–24.9 kg/m**^**2**^												
Infrequent	Ref		0.17		Ref		0.15		Ref		0.013	
< 20 g/day	0.78	0.65–0.95			0.96	0.72–1.27			0.67	0.52–0.86		
20–39 g/day	0.75	0.58–0.98			1.14	0.74–1.78			0.61	0.44–0.83		
≥ 40 g/day	0.96	0.71–1.29			1.65	0.99–2.74			0.78	0.55–1.11		
**BMI ≥ 25 kg/m**^**2**^												
Infrequent	Ref		0.028		Ref		0.018		Ref		0.16	
< 20 g/day	0.80	0.57–1.11			0.69	0.40–1.19			0.78	0.52–1.17		
20–39 g/day	0.63	0.39–1.04			0.26	0.08–0.86			0.76	0.44–1.32		
≥ 40 g/day	0.61	0.34–1.11			0.45	0.13–1.52			0.68	0.36–1.32		

Outcomes were CKD (composite outcome of eGFR decline and/or new-onset of proteinuria) and eGFR decline and new-onset of proteinuria, respectively.

*CKD* chronic kidney disease, *eGFR* estimated glomerular filtration rate, *BMI* body mass index, *CI* confidence interval, *HR* hazard ratio.

^a^Multivariable adjustment included age, sex, eGFR, hypertension, diabetes mellitus, hyper lipidemia, smoking status.

^b^*P* trend was derived from Cox proportional hazards regression models by treating alcohol consumption as a continuous linear term.

^c^*P* interaction was derived by using a likelihood ratio test from models with and without the cross-product term of alcohol category (Infrequent, light, moderate, and heavy drinkers) and risk factor in the multivariable-adjusted model.

## Discussion

In this study, we demonstrated that light drinking was associated with a lower risk of CKD, and this association was modified by the BMI status. When the BMI was low, alcohol consumption was associated with a higher risk of CKD. On the contrary, when the BMI was high, even heavy alcohol consumption was associated with a lower risk of CKD. These results suggest that a high BMI counteracts the harmful effects of CKD risks associated with alcohol consumption. Only a few studies have focused on the effect of BMI on the association between alcohol consumption and CKD development^21^ and, to the best of our knowledge, this is the first study to examine the association stratified by BMI.

Our findings on the dose–response association between alcohol consumption and CKD development are partially consistent with those of previous studies. A recent systematic review and two large prospective cohort studies showed that alcohol consumption was associated with a lower risk of CKD regardless of the amount of alcohol consumption^10,22,23^. Contrarily, in a large prospective study in Japan, only the light alcohol consumption group (< 20 g/day) was associated with a lower risk of CKD^11^. This discrepancy may be due to differences in the background of the target population. Previous epidemiological studies have reported that heavy alcohol consumption was associated with a higher incidence of HT and DM, which are known risk factors for CKD, but the relationship between alcohol consumption and their incidences was weakened in those with a higher BMI^14–20^. However, minimal evidence is available on the association between alcohol consumption and the risk of CKD, considering the effect of factors such as body mass on alcohol tolerance.

There are several possible mechanisms by which alcohol consumption may affect renal function, including protective^10^ and harmful effects^24,25^. Regarding protective effects, clinical studies have shown that moderate alcohol intake prevents atherosclerosis via alcohol-induced changes in lipid profile and inflammation^26^. Besides, animal studies have reported that low concentrations of alcohol protect podocytes, one of the key structures in the kidney, via acetaldehyde dehydrogenase and 20-hydroxyeicosatetraenoic acid^27^. Furthermore, alcohol consumption has been reported to improve insulin sensitivity^28,29^. Another animal study reported that podocytes are insulin sensitive and that this insulin sensitivity is important for maintaining the glomerular filtration barrier^30^. Regarding harmful effects, excessive alcohol consumption has been reported to have a negative effect on the risk of atherosclerosis^31^. Moreover, animal studies have reported that a high ethanol concentration significantly increases superoxide production in podocytes, resulting in increased oxidative stress and damage to podocytes^27^. In addition, excessive alcohol consumption can cause liver damage, cirrhosis, and secondary renal damage^32,33^. These mechanisms suggest that the harmful effects on renal function are more likely to occur when alcohol concentrations are high or when aldehydes, the intermediate metabolic products of alcohol, accumulate. These harmful effects are enhanced when alcohol is consumed beyond an individual’s capacity; BMI is one of the factors that determine this capacity^34,35^. In addition to body mass, genetic variations in the alcohol metabolism capacity should be considered in people with East Asian ancestry, including the Japanese populations. Mutations in the gene encoding the aldehyde dehydrogenase 2 (*ALDH2*) enzyme reduce acetaldehyde metabolism, which predisposes the flushing response. Individuals with mutations in the *ALDH2* gene are likely to have a lower BMI and to consume less alcohol, while those without these mutations tend to have a higher BMI^36^. Therefore, we considered that these mutations could partly account for the mechanism regarding the hypothesis that a high BMI would increase alcohol tolerance. A high BMI is still associated with alcohol tolerance, and our study demonstrated encouraging results regardless of the underlying mechanisms.

Our study has several limitations. First, the statistical power of the low BMI group and the heavy drinker group is considered to be too small to support a relationship. The four categories based on drinking status and stratification by BMI biased the number of people in each category, resulting in lower statistical power. However, changing the definition of drinking status or the classification of BMI enabled us to increase the statistical power and show that the relationship between drinking and CKD risk is modified by BMI. Second, we could not distinguish between individuals who had never consumed alcohol, those who consumed alcohol previously, and those who consumed alcohol occasionally at baseline, with all the above categorized as infrequent drinkers. Since the infrequent alcohol consumption category may have included people who abstained from drinking due to ill health or pre-existing conditions^37^, we excluded people with a history of CVD, COPD, or liver disease who may have stopped drinking due to their health conditions, to account for reverse causality bias^38^. In addition, we conducted the sensitivity analysis using light drinkers as a reference category, which showed a consistent result for the main analysis. Third, this study defined the highest category of alcohol consumption as ≥ 40 g/day and the lowest category as < 20 g/day, which may not be sufficient to show a dose–response relationship between alcohol consumption and CKD. In previous studies examining the association between risk of CKD development and alcohol consumption, with the highest consumption category being 60 g/day or more, the association was U-shaped or J-shaped^13,39^. Conversely, when the highest consumption category was 30 or 40 g/day or more, the risk decreased in a negative linear manner^23,40^. In our study, overall, the association between alcohol consumption and CKD risk was U-shaped, but there was a dose-dependent increase in risk in the low BMI group, U-shaped in the normal BMI group as well, with a dose-dependent decrease in risk in the high BMI group. Low BMI may overestimate GFR, and true CKD cases might be missed due to the decreased muscle mass, but the results rather showed that the relationship between alcohol consumption and CKD risk increased in the low BMI group, suggesting that the main analysis has a bias toward the null. In addition, heavy drinkers in the low BMI group may have a chronic alcohol use disorder, which may partially explain the relationship with an increased CKD risk^41^. Although it should be validated in a wider range of settings, we believe that stratification by BMI, which partly reflects an individual's alcohol tolerance, will help examine the appropriate alcohol intake associated with CKD in this study. Fourth, we did not consider metabolizing enzymes such as alcohol dehydrogenase‐1B and ALDH2. Almost all of the study participants were ethnic Japanese, and people with an Asian ancestry may be more sensitive to alcohol than those with a Caucasian ancestry due to genetic differences in alcohol-metabolizing enzymes^42^, which may have affected their drinking habits^43^. However, genetic tests are not widely available for health examination in the general population, nor is it routinely performed for risk stratification. Further studies are needed to evaluate the association between alcohol consumption and CKD development, considering genetic variations. Fifth, not all participants underwent continuous health checkups, and they may not have been tracked in the process due to moving to another municipality or death. However, we believe that bias is unlikely to occur in our cohort due to (a) continuity in receiving annual checkups by most participants, (b) the similarity in frequency and duration of follow-up across groups divided by drinking status, and (c) the exclusion of individuals with pre-existing conditions. Sixth, drinking habits and BMI changed over time, and we did not consider the effects of time-course changes in exposure statuses^44^. However, the scope of this study was to examine the relationship between the baseline exposure status of drinking and the future incidence of CKD. Examining the association between time-updated exposure status and CKD risk was beyond our scope. Finally, this was an observational study; therefore, unmeasured confounding factors may exist, although we adjusted for clinically relevant confounders for CKD. However, the strength of the present study is that in our cohort, more than 50% of the people who were eligible for the specified health checkups had been receiving the checks since the program was started in 2008. This percentage is higher than the national average of 30 to 35% for residents in other areas who are covered by the national health insurance scheme^45^. Moreover, we have comprehensive lifestyle information, anthropometric measurements, and blood and urine test results for more than 10,000 participants, and these data are comparable to those of the National Database of Health Insurance Claims and the Specific Health Examination Database Open Data Japan, which is considered representative and meaningful^46^.

In conclusion, light alcohol consumption was associated with a lower risk of developing CKD, and our findings underscore the effect modification of the association between alcohol consumption and CKD development by BMI. With a low BMI, even light to moderate alcohol consumption may be associated with the risk of developing CKD, indicating that the effects of alcohol consumption are not generalized and are dependent on the individuals’ constitution, such as body mass. Further study is needed to investigate the interaction between alcohol consumption and BMI for risk of CKD development in other key factors such as ethnicities and age groups.

## Methods

### Participant selection

We conducted a cohort study, named the Prevention of Development of Chronic Kidney Disease—the PROMISE Study, to investigate the association between alcohol consumption and new-onset CKD. Eligible participants were those aged 40–74 years who underwent specific health checkups at Kumiai Welfare Hospital, Takayama City, Gifu Prefecture, Japan, from April 2008 to December 2016. In this study, a cohort was formed with participants enrolled in the residential national health insurance program. We obtained information regarding drinking and smoking habits, antihypertensive and diabetes medications, and medical history using self-administered questionnaires.

All participants were anonymous, and the study complied with the Declaration of Helsinki and the Ethical Guidelines for Epidemiological Research published by the Ministry of Education, Culture, Sports, Science and Technology, and the Ministry of Health, Labour and Welfare in Japan. The requirement for written informed consent was waived by the Institutional Review Board Committee of Nagoya University Hospital (approval number: [2007-004]) because the study participants did not undergo invasive procedures, and only existing clinical data were collected.

### Definition of alcohol consumption

Regarding alcohol consumption, the participants were asked how often and how much sake (the standard drink in Japan) they consume per day at baseline. Daily alcohol consumption was assessed in units of “gou” (a traditional Japanese unit of measurement) and subsequently converted to grams of alcohol per day. One unit (gou) of sake contains 20 g of alcohol and is equivalent to 500 mL of beer, 60 mL of whiskey, or 240 mL of wine and has been listed accordingly in the questionnaire used by the participants^46,47^. The following two questions were asked to confirm the drinking habit: “How often do you drink alcoholic beverages: every day/occasionally/ rarely or never?” and “How many units of sake do you drink on the days you drink: < 1 drink per day/1–2 drinks per day/2–3 drinks per day/ ≥ 3 drinks per day?” Total alcohol consumption (in grams per day) was calculated from questions on the unit of sake consumed per day. We labeled the four categories as infrequent drinkers, light drinkers (< 20 g/day), moderate drinkers (20–39 g/day), and heavy drinkers (≥ 40 g/day). Since it was not feasible to quantify the precise amount of alcohol consumed by participants who answered “occasionally,” the participants who answered “rarely or never” and “occasionally” were grouped into the “infrequent drinkers” category for this analysis as the reference category. Some participants in the infrequent drinkers category may have refrained from drinking due to ill health or pre-existing conditions^37^. Therefore, we excluded participants with a history of CVD, COPD, or liver diseases. In the sensitivity analysis, based on the questionnaire, the definition of alcohol consumption was divided based on the frequency of drinking or the amount of alcohol per consumption, which were (Rarely or never/Occasionally/Every day) and (Rarely or never/< 20 g/20–39 g/≥ 40 g), respectively.

### Outcomes

Outcomes were measured using data collected from each visit, even for individuals with longer intervals between visits. The outcome was CKD development, defined as a 25% decline in eGFR to less than 60 mL/min/1.73 m^2^ and/or a dipstick urinalysis protein score of 1+ or greater (equivalent to ≥ 30 mg/dL) during the follow-up period^48^. Moreover, the effects of alcohol consumption on eGFR decline or proteinuria were investigated separately. To clarify whether the effects of alcohol consumption on the risk of CKD can be modified by the BMI status, we examined the association between alcohol consumption and the risk of CKD stratified by BMI.

### Physical examination and laboratory data

Trained nurses measured the participants’ BP using a standard protocol. After overnight fasting, venous blood was collected to measure the fasting plasma glucose, HbA1c, TG, HDL-C, LDL-C, and creatinine levels. Proteinuria was assessed using the dipstick method. eGFR was calculated using the formula of the Japanese Society of Nephrology [194 × age (year) − 0.287 × serum creatinine (mg/dL) − 1.094 (× 0.739 if female)]^49^.

### Other covariates

Current smokers were defined as participants with any of the following habits: those who had been smoking for more than 6 months or more than 100 cigarettes in total and those who smoked in the last month. Others were categorized as non-smokers, including those who had a previous smoking habit. Height was measured to the nearest 0.1 cm using a stadiometer, and weight was measured in kilograms using a digital scale, with the participant wearing light clothing and no shoes. BMI was calculated by dividing the weight (in kilograms) by the square of the height (in meters). We divided BMI into three categories (< 18.5: low, 18.5–24.9: normal, and ≥ 25.0: high). For sensitivity analysis, BMI was divided into the tertile and quartile to categorize. HT was defined as any of the following: systolic BP ≥ 140 mmHg, diastolic BP > 90 mmHg, or use of antihypertensive medications^50^. DM was defined as a fasting glucose level of ≥ 126 mg/dL, HbA1c (National Glycohemoglobin Standardization Program [NGSP]) level of ≥ 6.5%, or receiving glucose-lowering therapy^51^. After measuring the HbA1c level (%) in blood using the standard method proposed by the Japanese Diabetes Society (JDS), the NGSP equivalent was calculated using the following formula: HbA1c (NGSP) = HbA1c (JDS) + 0.4. Dyslipidemia was defined as an LDL-C level ≥ 140 mg/dL, HDL-C level < 40 mg/dL, TG level ≥ 150 mg/dL, or the use of lipid-lowering medication^52^.

### Statistical analysis

Baseline characteristics are presented by category of alcohol consumption (infrequent, light, moderate, and heavy drinkers). The mean or median was calculated for continuous variables, and percentages were calculated for dichotomous variables. To examine the association between baseline alcohol consumption and CKD development, Cox proportional hazards models were employed to calculate the HRs and 95% CIs for each alcohol consumption category using infrequent drinkers as the reference category. Multivariable adjustments were performed as follows: age and sex (model 1) and age, sex, eGFR, BMI, presence of HT, dyslipidemia, DM, and smoking status (current vs. noncurrent) (model 2). The proportional hazards assumption was tested using scaled Schoenfeld residuals. (Supplementary Figure S2) P for trend was derived from Cox proportional hazards models by treating alcohol consumption as a continuous linear term. Incidence rates were estimated using the person-year approach. We incorporated the interaction term between alcohol consumption and BMI categories into multivariable-adjusted models to assess the effect modification by BMI. P for interaction was derived using a likelihood ratio test from models with and without the cross-product term of alcohol category (infrequent, light, moderate, and heavy drinkers) and risk factors in the multivariable-adjusted model. All statistical analyses were performed using Stata version 16.0 MP (Stata Corp, www.stata.com). The statistical significance was set at *P* < 0.05.

### Ethical standards

We conducted the study in accordance with the guidelines of the Declaration of Helsinki. The institutional ethics committee of Nagoya University Graduate School of Medicine has formally approved the conduct of this study (No. 2017-0004). Since the study data were provided anonymously, and the study participants did not receive any intervention, informed consent for study participation was not required.

## Supplementary Information

Supplementary Information.

## Data Availability

The data that support the findings of this study are available from the corresponding author upon reasonable request.
