# Supplementary material for: Effect of body mass index on the association between alcohol consumption and the development of chronic kidney disease
Source: Sci Rep. 2021 Oct 14;11:20440. doi: 10.1038/s41598-021-99222-y (PMC8516880; doi:10.1038/s41598-021-99222-y)
Supplement: Supplementary file 1 — Supplementary Information. [file 41598_2021_99222_MOESM1_ESM.pdf]

**Supplementary Information**

# Title: Effect of Body Mass Index on the Association Between Alcohol Consumption and the Development of Chronic Kidney Disease

Yusaku Hashimoto[1], Takahiro Imaizumi[1*], Sawako Kato[1], Yoshinari Yasuda[1], Takuji Ishimoto[1], Hiroaki Kawashiri[2], Akihiro Hori[3] and Shoichi Maruyama[1*]

Author affiliations

[1] Department of Nephrology, Nagoya University Graduate School of Medicine, Nagoya, Aichi, Japan

[2] Takayama City Hall Public Health Department, Takayama, Gifu, Japan

[3] Kumiai Kosei Hospital, Takayama, Gifu, Japan

[*]Corresponding authors

Shoichi Maruyama, M.D., Ph.D. & Takahiro Imaizumi M.D., Ph.D.

Department of Nephrology, Nagoya University Graduate School of Medicine

65 Tsuruma-cho, Showa-ku, Nagoya, Aichi 464-8550, Japan

Tel: +81-52-744-2192 Fax: +81-52-744-2209

Email: marus@med.nagoya-u.ac.jp

Email: imaizumi18@med.nagoya-u.ac.jp

| | New-onset of CKD | | | |
|---|---|---|---|---|
| | Multivariate model[a] | | | |
| | HR | 95% CI | P trend[b] | P interaction[c] |
| **Drinking frequency** | | | | 0.016 |
| BMI <18.5 kg/m$^2$ | | | 0.022 | |
| Rarely or never | Ref. | | | |
| Occasionally | 1.46 | 0.74–2.87 | | |
| Every day | 3.44 | 1.60–7.42 | | |
| BMI 18.5–24.9 kg/m$^2$ | | | 0.018 | |
| Rarely or never | Ref. | | | |
| Occasionally | 0.80 | 0.66–0.99 | | |
| Every day | 0.79 | 0.64–0.97 | | |
| BMI ≥25 kg/m$^2$ | | | 0.028 | |
| Rarely or never | Ref. | | | |
| Occasionally | 0.80 | 0.56–1.14 | | |
| Every day | 0.66 | 0.45–0.96 | | |
| **Amount of alcohol per consumption** | | | | 0.004 |
| BMI <18.5 kg/m$^2$ | | | 0.002 | |
| Rarely or never | Ref. | | | |
| <20 g | 1.23 | 0.62–2.44 | | |
| 20–39 g | 2.88 | 1.42–5.83 | | |
| ≥40 g | 3.77 | 1.48–9.59 | | |
| BMI 18.5–24.9 kg/m$^2$ | | | 0.17 | |
| Rarely or never | Ref. | | | |
| <20 g | 0.79 | 0.65–0.97 | | |
| 20–39 g | 0.76 | 0.61–0.96 | | |
| ≥40 g | 0.99 | 0.74–1.32 | | |
| BMI ≥25 kg/m$^2$ | | | 0.033 | |
| Rarely or never | Ref. | | | |
| <20 g | 0.89 | 0.62–1.28 | | |
| 20–39 g | 0.65 | 0.43–0.97 | | |
| ≥40 g | 0.68 | 0.39–1.18 | | |

Supplementary Table S1. Results of Cox proportional hazards models for the association between drinking frequency, the amount of alcohol per consumption, and development of CKD, as stratified by BMI

Outcomes were CKD (composite outcome of eGFR decline and/or new-onset of

proteinuria).

a Multivariable adjustment included age, sex, eGFR, hypertension, diabetes mellitus, hyper lipidemia, smoking status.

b P trend was derived from Cox proportional hazards regression models by treating alcohol consumption status as a continuous linear term.

c P interaction was derived by using a likelihood ratio test from models with and without the cross-product term of each alcohol category and risk factor in the multivariable-adjusted model.

CKD chronic kidney disease; eGFR estimated glomerular filtration rate; BMI body mass index; CI confidence interval; HR hazard ratio.

| Amount of alcohol consumption | CKD | |
| --- | --- | --- |
| | Multivariate model[a] | |
| | HR | 95% CI |
| BMI <18.5 $kg/m^2$ | | |
| Infrequent | 0.80 | 0.42–1.52 |
| <20 g/day | Ref. | |
| 20–39 g/day | 2.74 | 1.21–6.20 |
| ≥40 g/day | 2.56 | 0.96–6.80 |
| BMI 18.5–24.9 $kg/m^2$ | | |
| Infrequent | 1.28 | 1.05–1.55 |
| <20 g/day | Ref. | |
| 20–39 g/day | 0.96 | 0.74–1.25 |
| ≥40 g/day | 1.23 | 0.91–1.65 |
| BMI ≥25 $kg/m^2$ | | |
| Infrequent | 1.25 | 0.90–1.75 |
| <20 g/day | Ref. | |
| 20–39 g/day | 0.63 | 0.48–1.32 |
| ≥40 g/day | 0.77 | 0.42–1.41 |

Supplementary Table S2. Results of a Cox proportional hazards model stratified by BMI for the association between alcohol consumption and CKD development using light drinkers (<20 g/day) as reference.

Outcomes were CKD (composite outcome of eGFR decline and/or new-onset of proteinuria).

a Multivariable adjustment included age, sex, eGFR, hypertension, diabetes mellitus, hyper lipidemia, smoking status.

CKD chronic kidney disease; eGFR estimated glomerular filtration rate; BMI body mass index; CI confidence interval; HR hazard ratio.

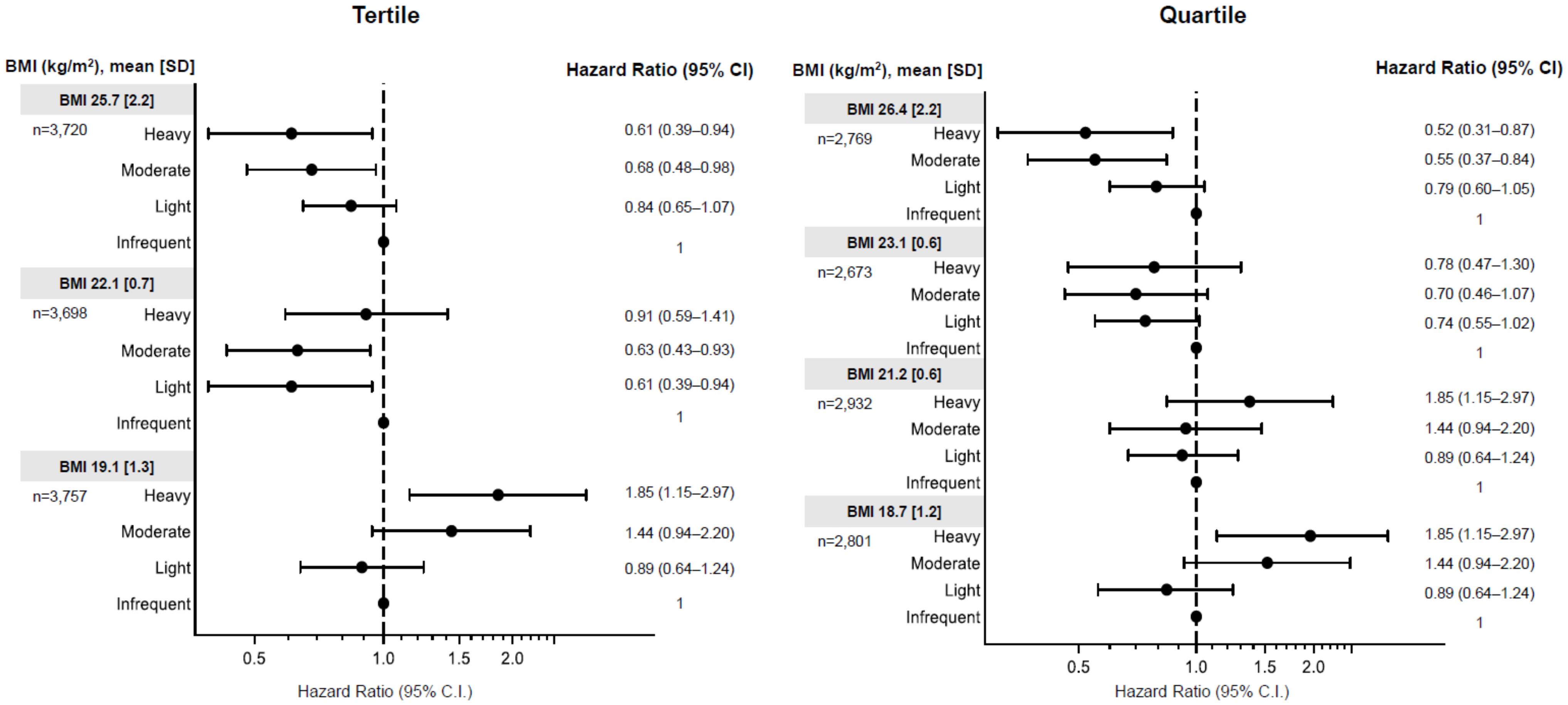


Supplementary Figure S1. Results of a Cox proportional hazards model stratified by BMI in tertile or quartiles of the association between alcohol consumption and CKD development. The categories of alcohol consumption status were labeled as infrequent, light (<20 g/day), moderate (20–39 g/day), and heavy (≥40 g/day). Outcomes were CKD (composite outcome of eGFR decline and/or new-onset of proteinuria). Multivariable adjustment included age, sex, eGFR, hypertension, diabetes mellitus, hyper lipidemia, smoking status.

CKD chronic kidney disease; eGFR estimated glomerular filtration rate; BMI body mass index; CI confidence interval; HR hazard ratio.

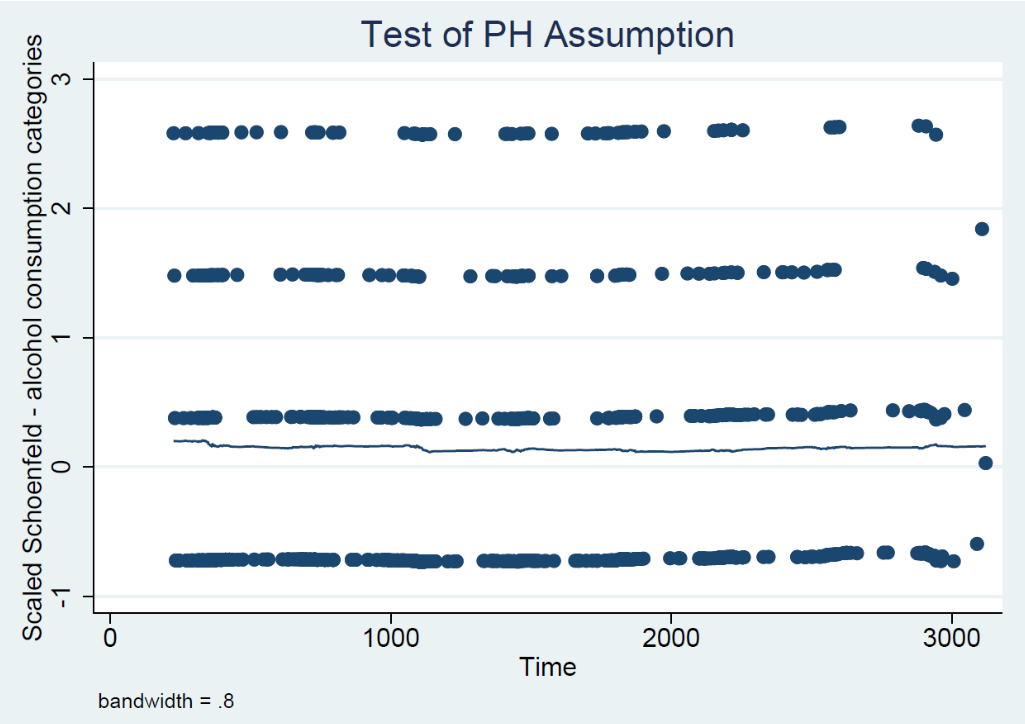


Supplementary Figure S2. Results of testing the assumption of proportional hazards using Schoenfeld residuals. r =-0.022, p =0.487.
